# Mid-to-long term mortality following surgical versus percutaneous coronary revascularization stratified according to stent subtype: An analysis of 6,682 patients with multivessel disease

**DOI:** 10.1371/journal.pone.0191554

**Published:** 2018-02-06

**Authors:** Shahzad G. Raja, Charles Ilsley, Fabio De Robertis, Rebecca Lane, Tito Kabir, Toufan Bahrami, Andre Simon, Aron Popov, Miles C. Dalby, Mark Mason, Richard Grocott-Mason, Robert D. Smith, M. Bilal Iqbal

**Affiliations:** 1 Department of Cardiac Surgery, Royal Brompton and Harefield NHS Foundation Trust, Harefield Hospital, Middlesex, United Kingdom; 2 Department of Cardiology, Royal Brompton and Harefield NHS Foundation Trust, Harefield Hospital, Middlesex, United Kingdom; 3 Department of Cardiology, Vancouver Island Health Authority, Royal Jubilee Hospital, Bay Street, Victoria, British Columbia, Canada; Azienda Ospedaliero Universitaria Careggi, ITALY

## Abstract

**Background:**

Studies comparing coronary artery bypass graft (CABG) and percutaneous coronary intervention (PCI) have largely been performed in the bare-metal stent (BMS) and first-generation drug eluting stent (F-DES) era. Second-generation DES (S-DES) have shown improved outcomes when compared to F-DES, but data comparing CABG with PCI using S-DES is limited. We compared mortality following CABG versus PCI for patients with multivessel disease and analyzed different stent types.

**Methods:**

A total of 6,682 patients underwent multivessel revascularization at Harefield Hospital, UK. We stratified CABG patients into single arterial graft (SAG) or multiple arterial grafts (MAG); and PCI patients into BMS, F-DES or S-DES groups. We analyzed all-cause mortality at 5 years.

**Results:**

4,388 patients had CABG (n[SAG] = 3,358; n[MAG] = 1,030) and 2,294 patients had PCI (n[BMS] = 416; n[F-DES] = 752; n[S-DES] = 1,126). PCI had higher 5-year mortality with BMS (HR = 2.27, 95% CI:1.70–3.05, p<0.001); F-DES (HR = 1.52, 95% CI:1.14–2.01, p = 0.003); and S-DES (HR = 1.84, 95% CI:1.42–2.38, p<0.001). This was confirmed in inverse probability treatment weighted analyses. When adjusting for both measured and unmeasured factors using instrumental variable analyses, PCI had higher 5-year mortality with BMS (Δ = 15.5, 95% CI:3.6,27.5, p = 0.011) and FDES (Δ = 16.5, 95% CI:6.6,26.4, p<0.001), but had comparable mortality with CABG for PCI with SDES (Δ = 0.9, 95% CI: -9.6,7.9, p = 0.844), and when exclusively compared to CABG patients with SAG (Δ = 0.4, 95% CI: -8.0,8.7, p = 0.931) or MAG (Δ = 4.6, 95% CI: -0.4,9.6, p = 0.931).

**Conclusions:**

In this real-world analysis, when adjusting for measured and unmeasured confounding, PCI with SDES had comparable 5-year mortality when compared to CABG. This warrants evaluation in adequately-powered randomized controlled trials.

## Introduction

Coronary artery bypass grafting (CABG) and percutaneous coronary intervention are established treatment options for patients with multivessel coronary disease. Previous studies have shown a survival benefit with CABG, but these studies have largely compared CABG to PCI with balloon angioplasty, bare-metal stents (BMS) and first-generation drug eluting stents (F-DES)[1–4]. Second-generation drug-eluting stents (S-DES) have thinner struts and more biocompatible polymers resulting in reduced inflammation and thrombogenicity[5]. Consequently, they have been shown to be more efficacious at reducing the risk of restenosis, stent thrombosis and death when compared to BMS and F-DES in broad clinical populations[6, 7]. However, there are limited data comparing CABG with PCI using S-DES. Randomized trials remain the gold standard for comparative effectiveness studies, but they can be limited by patient selection bias. Thus, it is important to confirm findings of randomized trials in the real world.

Whilst there has been increased interest in leveraging observational studies for comparative outcomes research, without robust and valid risk adjustment, findings from these studies may remain biased due to unmeasured factors. This is particularly the case when comparing PCI versus CABG for patients with multivessel disease. Non-randomized studies in his setting may not record specific coronary pathology or complexity of disease that would favour either revascularization strategy. Patient choices are also important and these may also contributory. PCI for multivessel disease is often reserved for the sicker and frailer patient or for those who may be poor candidates for surgery, particularly following multidisciplinary discussion. These factors may not be reliably captured by observational databases, and thus induce severe selection bias. In this study, we compared long-term survival following CABG and PCI in an all-comer real-world patient population with multivessel disease, and used analytical methods to address both measured and unmeasured confounding.

## Methods

This was a retrospective observational cohort study comparing long-term survival following CABG and PCI in an all-comer real-world patient population with multivessel disease. We used merged datasets from local British Cardiac Intervention Society (BCIS) database for PCI and National Adult Cardiac Surgery Audit (NACSA) database for CABG which contribute to the National Institute for Cardiovascular Outcomes Research (NICOR) databases. Although this is a retrospective analysis, all data was collected prospectively.

### Databases

The BCIS–NICOR and NACSA-NICOR databases collect data from all hospitals in UK that perform PCI and CABG, recording information about every procedure performed. Data are collected prospectively at each hospital, electronically encrypted and transferred online to a central database. Patients’ survival data is obtained by linkage of patients' National Health Service (NHS) numbers to the Office of National Statistics (ONS), which records the date of death for all patients.

### Population study and design

We examined an observational cohort of consecutive patients treated with PCI and CABG between 2004–2015 at Harefield Hospital, Middlesex, UK. Patient and procedural details were recorded at the time of the procedure. Initially, we identified 6,044 patients who had CABG (5,257 patients with multivessel disease) and 14,923 patients had PCI (7,149 patients with multivessel disease). We only included patients with multivessel disease and who underwent multi-vessel revascularization. Of these, 6,682 patients were included in the final analysis (4,388 patients in the CABG group and 2,294 patients in the PCI group).

### Definitions and clinical outcomes

A diseased coronary artery was defined as any epicardial vessel with a stenosis >70%. Multi-vessel disease was defined as stenosis >70% in ≥2 epicardial coronary arteries. The primary outcome was all-cause mortality at 5 years. For the CABG cohort, single arterial grafting (SAG) was defined as the use of a left internal mammary artery (LIMA) and saphenous vein graft(s) SVG(s); and multiple arterial grafting (MAG) was defined as the use of LIMA graft and either the use of right internal mammary artery (RIMA) and/or radial artery (RA) grafts. The use of SVGs was permitted in the MAG group.

### Ethics

All patient identifiable information was removed prior to database merging and analysis. Because this analysis was performed on anonymysed data from mandatory audit, the local ethics committee advised us that ethical approval was not required.

### Statistical analyses

Patients were divided into CABG and PCI groups. The CABG group was further sub-divided into SAG and MAG groups and the PCI groups was further subdivided into BMS, F-DES and S-DES groups. Non-categorical variables in our dataset had a skewed distribution, and thus were summarized using median (lower and upper quartiles) and compared using the Mann-Whitney U-test. Categorical variables were expressed as percentages and compared using the Z-test. All statistical analyses were performed using MedCalc v12.1 (MedCalc Software, Ostend, Belgium) and R (Foundation for Statistical Computing, Vienna, Austria). Statistical significance was established at p<0.05 (2-tailed) for all tests.

#### (a) Multivariate models for mortality

To determine independent predictors for mortality, Cox proportional hazards regression models were used to provide adjusted hazard ratios (HRs) with 95% confidence intervals (CIs). The proportional hazards assumption was tested and verified with Schoenfeld residuals. To guide selection of significant variables for the final multivariable models for each outcome, we initially adjusted for age, sex, diabetes, previous MI, previous PCI, hypertension, renal disease, previous cerebrovascular accident, peripheral vascular disease, smoking, severe LV function (LVEF≤30%), pre-procedural intra-aortic balloon pump (IABP) use, cardiogenic shock, stable presentation, presence of left main-stem (LMS) disease and year of procedure. The significant predictors from this were included in the final model with “PCI” as a forced-in variable. The following variables were included in the final multivariable model: age, sex, diabetes, renal disease, peripheral vascular disease, IABP use, cardiogenic shock, LMS disease and PCI. In this way, the number of variables was limited to 1 per ≥ 20 events to prevent over-fitting of the model.

#### (b) Inverse probability treatment weighted (IPTW) analysis

To further account for measured confounding, we performed IPTW analyses. In contrast to propensity-matched analyses which often result in a reduction of the population analyzed, this propensity score method utilizes the total study cohort in the final analysis. To derive propensity scores (*PS*_*i*_), a logistic regression model was fit for PCI to patient demographics, clinical, anatomical and procedural variables. The discriminatory power of the propensity score models were assessed using the receiver operator curve (ROC) analysis (c-statistic). *PS*_*i*_ were determined for all patients as above. IPTW analysis uses weights based on the *PS*_*i*_ to create a population in which the distribution of measured baseline covariates is independent of treatment assignment. These weights (*IPTW*_*i*_) were assigned to each patient with *IPTW*_*i*_ = 1/*PS*_*i*_ for treated patients (PCI); and *IPTW*_*i*_ = 1/(1-*PS*_*i*_) for control patients (CABG)[8]. Finally, Cox proportional hazards regression models were used to determine the effect of PCI vs. CABG incorporating significant covariates identified from the Cox multivariate models and *IPTW*_*i*_ in the final model (double-robust model).

#### (c) Instrumental variable (IV) analysis

IV analysis is an econometric method used to remove the effects of hidden bias in observational studies[9]. An IV has 2 key characteristics: (a) it is highly correlated with the treatment and (b) does not independently affect the outcome, other than via its effects through the treatment, so that it is not associated with measured or unmeasured variables. The enrollment year can serve as an effective IV[10], and we demonstrated this to be the case within our study. The enrollment year was not associated with 5-year mortality in both unadjusted models (HR = 0.99, 95% CI: 0.97–1.02, p = 0.597) and fully adjusted models (HR = 0.98, 95% CI: 0.94–1.02, p = 0.346). The F_statistic_ for the enrollment year as an IV was 49.9 (values < 10 are characteristic of weak IV). We initially performed adjusted linear regression, adjusting for the same covariates as in the Cox proportional hazards models (as above). An adjusted IV analysis was then performed using a simultaneous 2-stage least-squares regression approach. The first stage of this approach predicts the receipt of PCI (treatment) as a linear function of enrollment year (the instrument) and other observed covariates. In the second stage, clinical outcomes are regressed on the predicted probability of PCI use, derived from the first stage, along with the same measured confounders. In this way, the 2-stage least-squares approach to IV analysis generates consistent estimates of treatment effects that are unrelated to variations in treatment selection.

## Results

### Baseline population and procedural characteristics

We analyzed 6,682 patients who underwent multivessel revascularization for multivessel coronary disease. Of this patient population, 4388 patients were treated with CABG and 2,294 patients were treated with PCI (Table 1). Patients undergoing CABG were likely to be older, have diabetes, cerebrovascular disease and LMS disease. Patients undergoing PCI were more likely to be female, have renal disease, severe LV impairment, present with acute coronary syndrome and have pre-procedural cardiogenic shock. Of the patient treated with CABG, 3358 patients were treated with SAG and 1030 patients were treated with MAG (Table 2). Patients undergoing CABG with SAG were older, more likely to be female, have renal disease, peripheral vascular disease, severe LV impairment and present with acute coronary syndrome. Of patients treated with PCI, 416 patients were treated with BMS, 752 patients were treated with F-DES and 1126 patients were treated with S-DES (Table 3).

**Table 1:** Baseline demographic and procedural characteristics for total study population comparing CABG and PCI cohorts.

	Total (n = 6682)	CABG (n = 4388)	PCI (n = 2294)	p value
**Year of procedure**				
2004–2008	36.1	35.6	37.1	0.225
2009–2015	63.9	64.4	62.9	0.225
**Clinical factors**				
Age (years)	65(57,74)	66(58,74)	65(57,74)	0.012
Female	19.5	18.3	22.0	<0.001
PVD	8.1	10.6	2.3	<0.001
Renal disease *	2.8	2.2	4.4	<0.001
Previous CVA	5.1	6.3	2.2	<0.002
Previous MI	41.4	44.0	36.3	<0.001
Previous revascularization	29.9	17.1	54.6	<0.001
Diabetes	25.2	27.2	21.0	<0.001
Hypertension	73.4	80.2	57.4	<0.001
Smoking ^†^	12.6	10.6	17.6	<0.001
Severe LV function (EF≤30%)	5.2	4.7	7.0	0.002
**Presentation**				
Stable	60.0	62.9	54.5	<0.001
CCS	3(2,3)	3(2,3)	3(2,3)	0.913
CCS 1–2	49.5	49.7	49.1	0.688
CCS 3–4	50.5	50.3	50.9	0.688
NYHA	2(1,3)	2(2,3)	2(1,3)	<0.001
NYHA 1–2	69.4	68.9	70.3	0.270
NYHA 3–4	30.6	31.1	29.7	0.270
Cardiogenic shock	2.1	0.8	4.6	<0.001
Intubated	1.1	0.3	2.6	<0.001
IABP use ^#^	4.8	7.8	2.8	<0.001
**Coronary anatomy** ^§^				
LMS	25.9	28.8	20.4	<0.001
Number of diseased vessels	3(2,3)	3(2,3)	2(2,3)	<0.001
Number of vessels treated	2(2,3)	2(2,3)	2(2,2)	<0.001

Abbreviations: CVA, cerebrovascular accident; MI, myocardial infarction; PVD, peripheral vascular disease; IABP, intra-aortic balloon pump; LMS, left main stem artery; LV, left ventricle; NYHA, New York Heart Association; and CCS, Canadian Cardiovascular Society. Discrete variables are presented as percentages and compared using the Z-test (2-tailed); Continuous data presented as medians (25% IQ, 75% IQ) and compared using the Mann-Whitney U-test (2-tailed).

* Renal disease was defined as serum creatinine >150mmol/l or renal replacement therapy.

^†^ Smoking was defined as smoking of ≥1 cigarettes/day and had smoked in the month preceding PCI/CABG;

^§^ A diseased epicardial coronary vessel was defined as having a >70% coronary stenosis by visual estimation;

^#^ IABP insertion was either before or during procedure. Data with regards to timing of IABP insertion was not available.

**Table 2:** Baseline demographic and procedural characteristics for the CABG cohort.

	Total (n = 4388)	SAG (n = 3358)	MAG (n = 1030)	p value
**Year of procedure**				
2004–2008	35.6	39.4	23.0	<0.001
2009–2015	64.4	60.6	77.0	
**Clinical factors**				
Age (years)	66(58,74)	69(61,75)	62(55,68)	<0.001
Female	18.3	20.1	12.3	<0.001
PVD	10.6	11.4	7.8	0.001
Renal disease *	2.2	2.5	1.1	0.006
Previous CVA	6.3	7.2	3.5	<0.001
Previous MI	44.0	44.2	43.5	0.710
Previous revascularization	17.1	16.4	19.2	0.039
Diabetes	27.2	30.0	18.1	<0.001
Hypertension	80.2	80.8	78.1	0.055
Smoking ^†^	10.6	10.2	12.0	0.100
Severe LV function (EF≤30%)	4.7	5.4	2.7	<0.001
**Presentation**				
Stable	62.9	62.0	65.9	0.023
CCS	3(2,3)	3(2,3)	3(2,3)	0.234
CCS 1–2	50.3	49.2	54.2	0.005
CCS 3–4	49.7	50.8	45.8	0.005
NYHA	2(2,3)	2(2,3)	2(2,2)	<0.001
NYHA 1–2	68.9	66.8	75.8	<0.001
NYHA 3–4	31.1	33.2	24.2	<0.001
Cardiogenic shock	0.8	0.9	0.6	0.333
Intubated	0.3	0.4	0.2	0.417
IABP use ^#^	7.8	8.5	4.9	0.009
**Coronary anatomy** ^§^				
LMS	28.8	28.4	30.1	0.292
Number of diseased vessels	3(2,3)	3(2,3)	3(2,3)	0.314
Number of vessels treated	2(2,3)	3(2,3)	3(2,3)	<0.001

Discrete variables are presented as percentages and compared using the Z-test (2-tailed); Continuous data presented as medians (25% IQ, 75% IQ) and compared using the Mann-Whitney U-test (2-tailed). All abbreviations are as for Table 1.

**Table 3:** Baseline demographic and procedural characteristics for the PCI cohort.

	Total (n = 2294)	BMS (n = 416)	F-DES (n = 752)	S-DES (n = 1126)	p value
**Year of procedure**					
2004–2008	37.1	54.8	75.0	5.2	<0.001
2009–2015	62.9	45.2	25.0	94.8	<0.001
**Clinical factors**					
Age (years)	66(57,64)	68(60,77)	65(57,73)	66(57,65)	<0.001
Female	22.0	18.8	23.1	22.4	0.212
PVD	2.3	5.0	2.5	1.5	0.001
Renal disease *	4.4	6.8	2.6	4.5	0.011
Previous CVA	2.2	3.1	2.3	1.8	0.366
Previous MI	36.3	41.4	35.0	35.4	0.062
Previous revascularization	54.6	49.6	31.8	71.4	<0.001
Diabetes	21.0	18.6	21.9	21.3	0.447
Hypertension	57.4	53.8	58.5	53.2	0.152
Smoking ^†^	17.6	18.0	14.0	19.1	0.056
Severe LV function (EF≤30%)	7.0	8.7	5.8	7.2	0.395
**Presentation**					
Stable	54.5	51.9	69.1	45.7	<0.001
CCS	3(2,3)	2(2,3)	2(2,3)	2(2,4)	0.002
CCS 1–2	49.1	52.1	52.9	46.2	0.016
CCS 3–4	50.9	47.9	47.1	53.8	0.016
NYHA	2(1,3)	2(1,3)	2(1,3)	1(1,3)	<0.001
NYHA 1–2	70.3	71.1	73.9	68.1	0.043
NYHA 3–4	29.7	28.9	26.1	31.9	0.043
Cardiogenic shock	4.6	8.6	2.5	4.5	<0.001
Intubated	2.6	4.6	0.7	3.2	<0.001
IABP use ^#^	2.8	7.9	3.9	4.6	0.007
**Coronary anatomy** ^§^					
LMS	20.4	32.5	17.3	17.9	<0.001
Number of diseased vessels	2(2,3)	2(2,3)	2(2,3)	2(1,3)	<0.001
Number of vessels treated	2(2,2)	2(2,2)	2(2,2)	2(2,2)	0.015

Discrete variables are presented as percentages and continuous data presented as medians (25% IQ, 75% IQ). All comparisons have been made using ANOVA test (2-tailed). All abbreviations are as for Table 1.

### Unadjusted mortality

The unadjusted mortality rates were greater for PCI when compared to CABG at 30 days (4.8% vs. 2.1%, p<0.001); 1 year (7.8% vs. 4.0%, p<0.001); 3 years (12.0% vs. 7.0%, p<0.001); and 5 years (15.2% vs. 9.4%, p<0.001). In the CABG cohort, unadjusted mortality rates were greater for SAG when compared to MAG at 30 days (2.5% vs. 0.9%, p<0.001); 1 year (4.7% vs. 1.6%, p<0.001); 3 years (8.2% vs. 2.9%, p<0.001); and 5 years (11.2% vs. 3.5%, p<0.001). In the PCI cohort, unadjusted mortality rates were greater for BMS when compared to F-DES at 30 days (9.9% vs. 1.9%, p<0.001); 1 year (13.7% vs. 5.1%, p<0.001); 3 years (20.7% vs. 11.0%, p<0.001); and 5 years (26.9% vs. 16.4%, p<0.001); and when also compared to S-DES at 30 days (9.9% vs. 4.9%, p<0.001); 1 year (13.7% vs. 7.4%, p<0.001); 3 years (20.7% vs. 9.5%, p<0.001); and 5 years (26.9% vs. 10.1%, p<0.001). When comparing F-DES with S-DES, unadjusted mortality rates were greater with S-DES at 30 days (1.9% vs. 4.9%, p = 0.001) and 1 year (5.1% vs. 7.4%, p = 0.045); similar at 3 years (11.0% vs. 9.5%, p = 0.280); but lower with SDES at 5 years (16.4% vs. 10.1%, p<0.001)

### Adjusted mortality

#### (a) All PCI versus all CABG

When we performed adjusted analyses, and compared all PCI with all CABG, PCI was associated with higher mortality at 5 years (HR = 1.76, 95% CI: 1.46–2.12, p<0.001). These findings were confirmed when adjusting for measured confounders using IPTW analyses (HR = 1.98, 95% CI: 1.71–2.29, p<0.001). However, when adjusting for both measured and unmeasured confounders using IV analyses PCI and CABG had comparable mortality at 5 years (Δ = 2.9, 95% CI: -4.9–10.8, p = 0.463).

#### (b) Stratified analyses for stent type versus CABG

When examining 5-year mortality, stratified analyses found that PCI with BMS, F-DES and S-DES was associated with higher mortality when comparing to all CABG, CABG (SAG) and CABG (MAG). These findings were consistent when using standard Cox regression analysis and when adjusting for measured confounders using IPTW methods. When adjusting for measured and unmeasured confounders, compared to all CABG, PCI was still associated with higher mortality when using BMS (Δ = 15.5, 95% CI: 3.6–27.5, p = 0.011) and F-DES (Δ = 16.5, 95% CI: 6.6–26.4, p<0.001). However, PCI with S-DES had comparable mortality when compared to CABG (Δ = 0.9, 95% CI: -9.6–7.9, p = 0.844). Similarly, when comparing PCI to CABG with SAG, PCI was associated with higher mortality when using BMS (Δ = 23.6, 95% CI: 4.3–42.9, p = 0.017) and F-DES (Δ = 20.7, 95% CI: 9.5–31.8, p<0.001). However, PCI with S-DES had comparable mortality when compared to CABG (SAG) (Δ = 0.4, 95% CI: -8.0–8.7, p = 0.931). When comparing PCI to CABG with MAG, PCI was associated with higher mortality when using BMS (Δ = 69.0, 95% CI: 40.0–98.0, p<0.001) and F-DES (Δ = 14.7, 95% CI: 6.4–22.9, p<0.001). However, PCI with S-DES had comparable mortality when compared to CABG with MAG (Δ = 4.6, 95% CI: -0.4–9.6, p = 0.072). A summary of results obtained using the different adjustment methods are shown in Table 4.

**Table 4:** Summary of adjusted outcomes comparing PCI with CABG using different adjustment methods.

	PCI (n)	CABG (n)	Multivariable analyses		IPTW analyses		IV analyses	
**30-DAY MORTALITY**								
**Total study cohort**								
All PCI vs. All CABG	2294	4388	HR = 1.53 (1.05,2.22)	p = 0.025	HR = 2.57 (1.95,3.39)	p<0.001	Δ = 4.0 (-0.1,8.0)	p = 0.051
**Stent type vs. All CABG**								
BMS vs. All CABG	416	4388	HR = 2.30 (1.31,4.04)	p = 0.004	HR = 5.04 (3.48,7.30)	p<0.001	Δ = -6.0 (-21.9,9.8)	p = 0.454
DES vs. All CABG	1878	4388	HR = 1.59 (1.06,2.37)	p = 0.024	HR = 1.93 (1.41,2.64)	p<0.001	Δ = -0.3 (-3.8,3.3)	p = 0.880
F-DES vs. All CABG	752	4388	HR = 0.88 (0.40,1.94)	p = 0.749	HR = 0.98 (0.56,1.73)	p = 0.957	Δ = 0.4 (-7.1,8.0)	p = 0.909
S-DES vs. All CABG	1126	4388	HR = 2.02 (1.31–3.09)	p = 0.001	HR = 2.48 (1.77,3.48)	p<0.001	Δ = -0.2 (-2.6,2.1)	p = 0.848
**Stent type vs. SAG**								
BMS vs. SAG	416	3358	HR = 2.09 (1.18,3.68)	p<0.001	HR = 4.62 (3.18,6.71)	p<0.001	Δ = 0.3 (-18.6,19.2)	p = 0.973
DES vs. SAG	1878	3358	HR = 1.44 (0.95,2.17)	p = 0.084	HR = 1.72 (1.25,2.37)	p = 0.001	Δ = -2.4 (-6.5,1.6)	p = 0.244
F-DES vs. SAG	752	3358	HR = 0.84 (0.38,1.87)	p = 0.673	HR = 0.88 (0.49,1.56)	p = 0.667	Δ = 1.2 (-6.9,9.3)	p = 0.778
S-DES vs. SAG	1126	3358	HR = 1.81 (1.17–2.80)	p = 0.008	HR = 2.23 (1.58–3.14)	p<0.001	Δ = 0.7 (-3.9,5.4)	p = 0.755
**Stent type vs. MAG**								
BMS vs. MAG	416	1030	HR = 3.46 (1.08,11.15)	p = 0.032	HR = 9.71 (4.71,20.38)	p<0.001	Δ = 9.0 (-5.8,23.8)	p = 0.233
DES vs. MAG	1878	1030	HR = 2.36 (0.82,6.79)	p = 0.112	HR = 4.24 (2.11,8.54)	p<0.001	Δ = 1.8 (-10.8,14.3)	p = 0.780
F-DES vs. MAG	752	1030	HR = 1.46 (0.36,5.87)	p = 0.593	HR = 1.77 (0.74,4.21)	p = 0.202	Δ = 0.4 (-2.9,3.8)	p = 0.795
S-DES vs. MAG	1126	1030	HR = 3.66 (1.21,11.08)	p<0.001	HR = 5.45 (2.67,11.14)	p<0.001	Δ = -0.4 (-4.1,3.4)	p = 0.844
**1-YEAR MORTALITY**								
**Total study cohort**								
All PCI vs. All CABG	2294	4388	HR = 1.68 (1.27,2.22)	p<0.001	HR = 2.10 (1.70,2.59)	p<0.001	Δ = -2.3 (-7.8,3.2)	p = 0.417
**Stent type vs. All CABG**								
BMS vs. All CABG	416	4388	HR = 2.08 (1.33,3.24)	p<0.001	HR = 3.58 (2.60,4.80)	p<0.001	Δ = 20.7 (12.2,29.2)	p<0.001
DES vs. All CABG	1878	4388	HR = 1.73 (1.29,2.33)	p<0.001	HR = 1.76 (1.39,2.22)	p<0.001	Δ = -2.3 (-7.8,3.2)	p = 0.417
F-DES vs. All CABG	752	4388	HR = 1.54 (0.96,2.46)	p = 0.073	HR = 1.41 (0.99,2.01)	p = 0.058	Δ = 6.3 (-0.3,12.9)	p = 0.061
S-DES vs. All CABG	1126	4388	HR = 1.92 (1.38–2.67)	p<0.001	HR = 1.95 (1.50,2.55)	p<0.001	Δ = 0.4 (-2.7,3.5)	p = 0.786
**Stent type vs. SAG**								
BMS vs. SAG	416	3358	HR = 1.89 (1.21,2.96)	p<0.001	HR = 3.20 (2.35,4.36)	p<0.001	Δ = 38.7 (24.2,53.2)	p<0.001
DES vs. SAG	1878	3358	HR = 1.58 (1.17,2.15)	p = 0.003	HR = 1.56 (1.23,1.98)	p<0.001	Δ = 6.2 (-5.0,17.5)	p = 0.278
F-DES vs. SAG	752	3358	HR = 1.42 (0.89,2.28)	p = 0.147	HR = 1.25 (0.87,1.79)	p = 0.227	Δ = 8.0 (0.7,15.3)	p = 0.033
S-DES vs. SAG	1126	3358	HR = 1.75 (1.25–2.45)	p = 0.001	HR = 1.75 (1.34–2.30)	p<0.001	Δ = 0.5 (-2.6,3.4)	p = 0.753
**Stent type vs. MAG**								
BMS vs. MAG	416	1030	HR = 3.00 (1.29,7.00)	p = 0.011	HR = 6.79 (3.82,12.11)	p<0.001	Δ = 20.6 (3.0,38.3)	p = 0.022
DES vs. MAG	1878	1030	HR = 2.85 (1.36,5.99)	p = 0.006	HR = 3.89 (2.30,6.59)	p<0.001	Δ = -5.9 (-23.1,11.3)	p = 0.503
F-DES vs. MAG	752	1030	HR = 3.10 (1.31,7.35)	p = 0.011	HR = 3.16 (1.74,5.73)	p<0.001	Δ = 3.6 (-1.5,8.7)	p = 0.168
S-DES vs. MAG	1126	1030	HR = 3.16 (1.44,6.92)	p = 0.004	HR = 4.16 (2.41,7.17)	p<0.001	Δ = -2.7 (-1.3,6.7)	p = 0.183
**3-YEAR MORTALITY**								
**Total study cohort**								
All PCI vs. All CABG	2294	4388	HR = 1.69 (1.39,2.06)	p<0.001	HR = 1.99 (1.67,2.35)	p<0.001	Δ = 0.7 (-2.7,4.1)	p = 0.668
**Stent type vs. All CABG**								
BMS vs. All CABG	416	4388	HR = 2.04 (1.47,2.83)	p<0.001	HR = 3.00 (2.35,3.83)	p<0.001	Δ = 19.8 (9.0,30.5)	p<0.001
DES vs. All CABG	1878	4388	HR = 1.63 (1.32,2.03)	p<0.001	HR = 1.74 (1.44,2.09)	p<0.001	Δ = 1.4 (-5.7,8.5)	p = 0.708
F-DES vs. All CABG	752	4388	HR = 1.50 (1.08,2.08)	p = 0.012	HR = 1.56 (1.22,2.00)	p<0.001	Δ = 16.5 (3.6,29.4)	p = 0.012
S-DES vs. All CABG	1126	4388	HR = 1.75 (1.36–2.24)	p<0.001	HR = 1.86 (1.48,2.34)	p<0.001	Δ = -2.5 (-14.0,9.1)	p = 0.676
**Stent type vs. SAG**								
BMS vs. SAG	416	3358	HR = 1.90 (1.37,2.63)	p<0.001	HR = 2.78 (2.17,3.57)	p<0.001	Δ = 22.2 (7.1,37.3)	p<0.001
DES vs. SAG	1878	3358	HR = 1.51(1.21,1.88)	p<0.001	HR = 1.58 (1.31,1.91)	p<0.001	Δ = -0.3 (3.7–3.1)	p = 0.846
F-DES vs. SAG	752	3358	HR = 1.40 (1.00,1.95)	p = 0.049	HR = 1.43 (1.10,1.84)	p = 0.006	Δ = 12.6 (2.9–22.2)	p = 0.012
S-DES vs. SAG	1126	3358	HR = 1.62 (1.25–2.09)	p<0.001	HR = 1.70 (1.35–2.15)	p<0.001	Δ = -1.4 (9.0–13.5)	p = 0.349
**Stent type vs. MAG**								
BMS vs. MAG	416	1030	HR = 2.95 (1.77,4.90)	p<0.001	HR = 5.16 (3.34,7.99)	p<0.001	Δ = 42.1 (18.9,65.2)	p<0.001
DES vs. MAG	1878	1030	HR = 2.64 (1.74,4.02)	p<0.001	HR = 3.26 (2.20,4.82)	p<0.001	Δ = 8.4 (-2.0,18.8)	p = 0.114
F-DES vs. MAG	752	1030	HR = 2.53 (1.53,4.19)	p<0.001	HR = 2.84 (1.84,4.37)	p<0.001	Δ = 8.2 (1.2,15.2)	p = 0.025
S-DES vs. MAG	1126	1030	HR = 2.66 (1.69,4.27)	p<0.001	HR = 3.36 (2.19,5.15)	p<0.001	Δ = -0.8 (5.6,4.0)	p = 0.745
**5-YEAR MORTALITY**								
**Total study cohort**								
All PCI vs. All CABG	2294	4388	HR = 1.76 (1.46,2.12)	p<0.001	HR = 1.98 (1.71,2.29)	p<0.001	Δ = 2.9 (-4.9,10.8)	p = 0.463
**Stent type vs. All CABG**								
BMS vs. All CABG	416	4388	HR = 2.27 (1.70,3.05)	p<0.001	HR = 2.97 (2.40,3.68)	p<0.001	Δ = 15.5 (3.6,27.5)	p = 0.011
DES vs. All CABG	1878	4388	HR = 1.65 (1.34,2.03)	p<0.001	HR = 1.72 (1.45,2.02)	p<0.001	Δ = 2.9 (-4.9,10.8)	p = 0.463
F-DES vs. All CABG	752	4388	HR = 1.52 (1.14,2.01)	p = 0.003	HR = 1.60 (1.30,1.97)	p<0.001	Δ = 16.5 (6.6,26.4)	p<0.001
S-DES vs. All CABG	1126	4388	HR = 1.84 (1.42–2.38)	p<0.001	HR = 1.84 (1.47,2.29)	p<0.001	Δ = -0.9 (-9.6,7.9)	p = 0.844
**Stent type vs. SAG**								
BMS vs. SAG	416	3358	HR = 2.11 (1.57,2.83)	p<0.001	HR = 2.75 (2.22,3.41)	p<0.001	Δ = 23.6 (4.3,42.9)	p = 0.017
DES vs. SAG	1878	3358	HR = 1.53 (1.24,1.89)	p<0.001	HR = 1.69 (1.35,2.11)	p<0.001	Δ = -1.9 (-18.0,14.1)	p = 0.815
F-DES vs. SAG	752	3358	HR = 1.42 (1.07,1.88)	p = 0.017	HR = 1.46 (1.19,1.80)	p<0.001	Δ = 20.7 (9.5,31.8)	p<0.001
S-DES vs. SAG	1126	3358	HR = 1.71 (1.32–2.22)	p<0.001	HR = 1.69 (1.35–2.11)	p<0.001	Δ = 0.4 (-8.0–8.7)	p = 0.931
**Stent type vs. MAG**								
BMS vs. MAG	416	1030	HR = 3.81 (2.25,6.46)	p<0.001	HR = 5.51 (3.72,8.16)	p<0.001	Δ = 69.0 (40.0,98.0)	p<0.001
DES vs. MAG	1878	1030	HR = 2.78 (1.76,4.41)	p<0.001	HR = 3.36 (2.35,4.80)	p<0.001	Δ = 6.2 (-15.3,27.7)	p = 0.574
F-DES vs. MAG	752	1030	HR = 2.64 (1.58,4.42)	p<0.001	HR = 3.02 (2.06,4.43)	p<0.001	Δ = 14.7 (6.4,22.9)	p<0.001
S-DES vs. MAG	1126	1030	HR = 3.36 (1.96,5.75)	p<0.001	HR = 3.38 (2.126,5.06)	p<0.001	Δ = 4.6 (-0.4,9.6)	p = 0.072

All adjusted ratios are referent to PCI. Highlighted boxes indicate comparable outcomes with PCI and CABG.

#### (c) Stratified analyses for DES type versus CABG

Having established that PCI with SDES had comparable outcomes with CABG in IV analyses, we sought to compare outcomes for PCI with CABG when stratified to different DES types. A total of 377 patients received paclitaxel-eluting stents (PES), 475 patients received sirolimus-eluting stents (SES), 286 patients received everolimus-eluting stents (EES) and 922 patients received zotarolimus-eluting stents (ZES). Thus the FDES cohort included PES and SES; and the SDES cohort included EES and ZES. When examining 5-year mortality using standard Cox regression and IPTW analyses, all types of DES were associated with worse outcomes when compared to CABG. However, when adjusting for measured and unmeasured confounders, PCI with PES and SES were still associated with worse outcomes, but PCI with EES and ZES were associated with comparable outcomes. A summary of results obtained using the different adjustment methods are shown in Table 5.

**Table 5:** Summary of adjusted outcomes comparing different DES types with CABG using different adjustment methods.

	PCI (n)	CABG (n)	Multivariable analyses		IPTW analyses		IV analyses	
**30-DAY MORTALITY**								
PES vs. CABG	377	4388	HR = 0.55 (0.08,3.94)	p = 0.554	HR = 0.53 (0.19,1.47)	p = 0.227	Δ = 0.7 (-2.2,3.6)	p = 0.640
SES vs. CABG	475	4388	HR = 0.88 (0.38,2.04)	p = 0.773	HR = 1.11 (0.57,2.16)	p = 0.752	Δ = 2.3 (-3.4,8.0)	p = 0.423
EES vs. CABG	286	4388	HR = 1.15 (0.50,2.64)	p = 0.744	HR = 1.76 (0.90,3.43)	p = 0.098	Δ = -2.2 (-8.1,3.4)	p = 0.471
ZES vs. CABG	922	4388	HR = 1.77 (1.18,2.66)	p = 0.006	HR = 2.75 (1.94,3.90)	p<0.001	Δ = 1.5 (-3.8,6.9)	p = 0.573
**1-YEAR MORTALITY**								
PES vs. CABG	377	4388	HR = 1.33 (0.54,3.24)	p = 0.536	HR = 0.79 (0.43,1.46)	p = 0.451	Δ = 1.6 (-2.4,5.5)	p = 0.436
SES vs. CABG	475	4388	HR = 1.41 (0.85,2.33)	p = 0.185	HR = 1.67 (1.12,2.50)	p = 0.013	Δ = 6.3 (-1.6,14.1)	p = 0.116
EES vs. CABG	286	4388	HR = 1.47 (0.85,2.54)	p = 0.175	HR = 1.75 (1.07,2.88)	p = 0.028	Δ = 1.1 (-6.8,8.9)	p = 0.792
ZES vs. CABG	922	4388	HR = 1.65 (1.21,2.27)	p = 0.002	HR = 2.01 (1.52,2.66)	p<0.001	Δ = 0.2 (-3.4,3.8)	p = 0.887
**3-YEAR MORTALITY**								
PES vs. CABG	377	4388	HR = 1.25 (0.67,2.36)	p = 0.486	HR = 1.32 (0.93,1.88)	p = 0.128	Δ = 5.3 (0.1,10.6)	p = 0.048
SES vs. CABG	475	4388	HR = 1.51 (1.05,2.17)	p = 0.026	HR = 1.77 (1.32,2.36)	p<0.001	Δ = 10.9 (0.5,21.2)	p = 0.039
EES vs. CABG	286	4388	HR = 1.36 (0.82,2.26)	p = 0.240	HR = 1.60 (1.00,2.47)	p = 0.053	Δ = 2.8 (-3.8,9.4)	p = 0.407
ZES vs. CABG	922	4388	HR = 1.77 (1.36,2.31)	p<0.001	HR = 1.90 (1.49,2.42)	p<0.001	Δ = 1.6 (-7.2,10.4)	p = 0.717
**5-YEAR MORTALITY**								
PES vs. CABG	377	4388	HR = 1.63 (1.03,2.55)	p = 0.036	HR = 1.54 (1.17,2.03)	p = 0.002	Δ = 12.3 (6.2,18.3)	p<0.001
SES vs. CABG	475	4388	HR = 1.54 (1.14,2.09)	p = 0.005	HR = 1.66 (1.29,2.12)	p<0.001	Δ = 16.5 (4.8,28.2)	p = 0.006
EES vs. CABG	286	4388	HR = 1.31 (0.79,2.17)	p = 0.298	HR = 1.59 (1.01,2.49)	p = 0.046	Δ = -3.9 (-11.3,3.5)	p = 0.777
ZES vs. CABG	922	4388	HR = 1.78 (1.38,2.29)	p<0.001	HR = 1.87 (1.49,2.36)	p<0.001	Δ = -1.4 (-11.2,8.4)	p = 0.867

All adjusted ratios are referent to PCI. Highlighted boxes indicate comparable outcomes with PCI and CABG.

## Discussion

This study compares long-term mortality following PCI and CABG for multivessel disease in the real world. It is the first study to provide stratified comparisons for PCI according to stent type with CABG according to number of arterial grafts, whilst adjusting for measured and unmeasured factors. The results of this study have shown that when using standard adjustment and propensity score methods to address measured confounders, PCI was associated with worse outcomes even when stratified to BMS, F-DES and S-DES and compared to CABG with SAG and MAG. However, when additionally addressing unmeasured factors, PCI with BMS and F-DES were still associated with worse outcomes, but PCI with S-DES had comparable outcomes with CABG.

Current guidelines for coronary revascularization in patients with multivessel disease state a class I indication for CABG[11, 12]. However, the use of PCI in the treatment of such patients has now been promoted from previous ESC guidance, from a class IIa to a class I (level of evidence B) in those patients with MVD and a ‘SYNergy between percutaneous intervention with TAXus and cardiac surgery's’ (SYNTAX) score ≤22[1, 11]. This is a consequence of advances in technology, with new generation drug-eluting stents (DES), adjunctive assessment tools (intravascular ultrasound and fractional flow reserve) and more potent anti-platelet agents. Furthermore, individual operators have gained increasing experience in dealing with complex PCI. However, in patients with multivessel disease, full risk stratification and active discussion with a multi-disciplinary ‘Heart Team’ are strongly recommended^11, 12^. It is imperative that the most appropriate revascularization modality taking into account patient baseline clinical and angiographic characteristics is chosen to provide favorable long-term outcomes for the patient.

Previous studies demonstrating a survival benefit of CABG over PCI for patients with multivessel disease have compared CABG with either balloon angioplasty, BMS and F-DES[1–4]. The introduction of DES represents a significant advance in interventional cardiology. The F-DES were associated with reduced restenosis and repeat revascularization rates compared to BMS[13–15] but there were concerns with regards to their long-term safety[16–18]. S-DES have been designed with new biocompatible polymer coatings, less toxic anti-proliferative drugs and thinner struts and have improved outcomes when compared to F-DES and BMS[5–7]. However, there are limited data comparing CABG with PCI in the S-DES era. Data from the New York State registries have shown a reduction in the gap between CABG and PCI with respect to mortality from the balloon angioplasty era[2] (40–50% reduction in mortality with CABG) to the BMS era[3] (24–35% reduction) to F-DES era[4] (20–29% reduction). This gap is further reduced with S-DES, as recently shown, where no significant difference in mortality was demonstrated with PCI with everolimus-eluting stents and CABG[19]. For CABG, results derived from observational analyses over the past decade suggests that using a second arterial graft improves intermediate and long-term outcomes compared with the use of a single arterial graft[20–25]. However, the Arterial Revascularization Trial (ART) comparing single versus bilateral mammary arterial grafts demonstrated no difference in 1 year outcomes[26], although the long-term results are awaited. A contemporary analysis comparing different stent types with CABG should stratify CABG patients into SAG and MAG if possible. To date, only one observational study has compared PCI with either BMS or DES with CABG with SAG and MAG, and found that CABG with MAG was associated with greatest survival[27].

Whilst randomized controlled trials remain the gold standard for comparative effectiveness studies, it is important to confirm findings trials in the real world patient population. This is particularly the case for DES as current use patterns have extended well beyond the original trial populations. Indeed, more than half of all DES implants are performed for “off-label” indications[28]. However, results from non-randomized studies may remain biased as standard adjustment methods, including propensity score methods only address measured confounders and residual confounding due to unmeasured factors will always exist[29]. IV analysis is an econometric method which is gaining popularity in observational outcomes research and can address confounding due to measured and unmeasured factors. This method has been used in comparative outcomes studies for DES versus BMS[10, 30]. Similar to randomization, an IV is related to treatment selection but not directly related to the outcome. Its occurrence creates a natural experiment and can overcome the effect of unmeasured confounders. To date, there are no real world analyses comparing PCI and CABG that have systematically addressed treatment selection bias.

Our analyses have shown that when adjusting for measured factors using standard adjustment and propensity score methods, PCI was associated with worse outcomes. These findings are consistent with previously reported analyses using propensity score methods. However, when adjusting for measured and unmeasured factors, IV analyses indicated that PCI with BMS and F-DES were still associated with higher mortality, but PCI with S-DES was associated with comparable mortality when compared to CABG (SAG or MAG). When we performed analyses comparing different types of DES, we found that both types of SDES (EES and ZES) had comparable mortality compared to CABG. It must be noted that whilst these subgroup analyses have demonstrated interesting findings, such analyses reduce the population studied and consequently the statistical power to detect differences.

A recent single centre retrospective analysis comparing PCI and CABG in 8,402 patients with multivessel disease found a strategy of CABG with SAG had comparable survival compared to PCI with BMS or DES. However, a strategy of CABG with MAG to be associated with improved survival when compared to PCI with BMS or DES[27]. These results do need to be interpreted with caution. Firstly, 77% of patients in the DES cohort in this study had F-DES, thus the results may not be applicable in the contemporary DES era. Secondly, whilst propensity-matching was used to adjust for measured confounders, unmeasured confounding was not addressed, and thus the results of this study may remain biased. Patients in the MAG cohort were a lower risk population when compared to the SAG cohort, i.e. were younger, less likely to be female, have renal disease, diabetes, congestive cardiac failure, previous myocardial infarction, lower left ventricular function, obstructive airways disease, three-vessel disease and with left main stem involvement. We also found that the MAG cohort was a lower risk population when compared to the SAG cohort. Thus it is highly likely that standard adjustment methods may still yield biased results owing to unmeasured factors. Our study provides a contemporary analysis and provides stratified comparisons for PCI according to stent type and CABG according to number of arterial grafts, whilst rigorously adjusting for both measured and unmeasured confounding. To date, there is only one randomized trial comparing CABG versus PCI with S-DES. The Randomized Comparison of Coronary Artery Bypass Surgery and Everolimus-Eluting Stent Implantation in the Treatment of Patients with Multivessel Coronary Artery Disease (BEST) trial compared PCI with S-DES (everolimus-eluting stent) and CABG in patients with multivessel disease[31]. The trial was stopped early due to slow enrolment. The results showed non-inferiority of PCI at 2 years but at 4.6 years, the primary end-point (composite of death, myocardial infarction and repeat revascularization) was greater with PCI, but this was driven by increased repeat revascularization events. Whilst the study was underpowered to detect differences in mortality, there were no differences in long-term mortality between PCI and CABG. The SYNTAX 2 trial has completed its recruitment and aims to compare CABG versus PCI with S-DES (Synergy stent, and everolimus-eluting stent with biodegradable polymer) in 450 patients with de novo 3 vessel coronary disease (excluding LMS disease)[32]. The results of this trial are eagerly awaited.

The main strength of this study is that it is the first to provide a systematic contemporary analysis of PCI versus CABG comparing different stents and surgical revascularization strategies whilst addressing both measured and unmeasured confounding. Data with regards to (a) complexity of coronary disease; (b) rationale for revascularization strategies (c) results of myocardial viability studies that may have favored one strategy over another; (d) patient choices; and (e) patient frailty status and robustness for surgery were not captured by the database. Theoretically these unmeasured factors should be address by the IV methods used in the analysis, but this study still has the limitations and the potential bias associated with non-randomization and thus residual confounding cannot be wholly excluded. Outcomes such as repeat revascularization and stroke were not available. These outcomes would be important when comparing PCI and CABG, particularly as repeat revascularization rates are greater in patients following PCI and stroke rates are greater following CABG. However, the use of all-cause mortality is considered a hard clinical endpoint in cardiovascular studies[33]. Nevertheless, the results of our study indicate that with the evolution of stent technology, the survival benefit gap between CABG and PCI has progressively become smaller, and that PCI and CABG have comparable mortality in S-DES era. Another strength of this study is that it compares outcomes in the real world. Whilst this may be considered “guideline free practice”, and thus a limitation to the study, given that most DES use in the real world are for off-label clinical indication, it is important to evaluate outcomes in the real world clinical practice. Given that this is a single centre study, the results of this study may not be generalizable and the results warrant confirmation in larger multicenter datasets and prospective randomized controlled trials.

## Conclusions

In this real-world analysis, when adjusting for measured and unmeasured confounding, PCI with BMS and F-DES was associated with greater long-term mortality, whilst PCI with SDES had comparable 5-year mortality when compared to CABG. This warrants evaluation in prospective adequately-powered randomized controlled trials.
